# Computational and statistical analyses of blood hemodynamic inside cerebral aneurysms for treatment evaluation of endovascular coiling

**DOI:** 10.1038/s41598-023-47867-2

**Published:** 2023-11-22

**Authors:** Rong Yang, Lian Yang, Golnar Ghane

**Affiliations:** 1 Shandong Cancer Hospital and Institute, Shandong First Medical University and Shandong Academy of Medical Sciences, Jinan, 250117 China; 2 Department of Medical Surgical Nursing, School of Nursing and Midwifery, Tehran University of Medical Sciences, Tehran, Iran

**Keywords:** Biomedical engineering, Mechanical engineering

## Abstract

Diagnosis of aneurysm and possibility of aneurysm rupture are crucial for avoiding brain hemorrhage. In this work, blood stream inside internal carotid arteries (ICAs) are simulated in diverse working conditions to disclose the importance of hemodynamic factors on the rupture of aneurysm. The main attention of this study is to investigate the role of hemodynamic on the aneurysm rupture. Statistical and computational methods are applied to investigate coiling porosity and blood hematocrit in 9 specific real ICA geometries. Response surface model (RSM) develops 25 runs to investigate all features of selected geometrical parameters and treatment factors. Computational fluid dynamic is used for the simulation of the blood stream in the selected aneurysms. The effects of sac section area and mean radius of parent vessel on blood hemodynamics are fully investigated. Hemodynamic factors are examined and compared at the peak systolic time instant, including pressure distributions, and velocity. Achieved results indicate that the increasing sac section area (from 36.6 to 75.4 mm^2^) results in 20% pressure reduction on the sac wall.

## Introduction

The development and progression of cardiovascular diseases are primarily attributed to the biomechanical interactions occurring between the blood flow and the vessel wall. Understanding the local flow mechanics and their association with disease evolution is a key focus of both experimental and computational research in blood hemodynamics^1,2^. However, describing the complex and diverse blood flow situations in diseased vessels, even without considering the coupled biophysical or biochemical processes driving disease progression, can be challenging^3,4^. Nevertheless, the "near-wall" region within the vessel plays a crucial role in these interactions, as it experiences the most intense couplings. In this region, the blood flow applies mechanical stresses on the vessel wall and regulates the local transport of reactive substances between the fluid domains and tissues. This aspect holds great significance, and the present work aims to elucidate this characteristic in the context of aneurysm rupture^5,6^.

An intracranial aneurysm (ICA) is a bulging or ballooning of a blood vessel in the brain. Endovascular coiling is a minimally invasive procedure used to treat ICA aneurysms by inserting small coils into the aneurysm to promote clotting and prevent blood flow into the aneurysm. Blood hemodynamics refer to the flow of blood through blood vessels, including ICA aneurysms. Evaluating blood hemodynamics inside ICA aneurysms is important for assessing the effectiveness of endovascular coiling. During the endovascular coiling procedure, coils are placed inside the aneurysm to promote clotting and prevent blood flow. Blood hemodynamics are then evaluated to determine if the coils are effectively blocking blood flow into the aneurysm. To further elaborate, blood hemodynamics inside ICA aneurysms can be evaluated using various imaging techniques such as Doppler ultrasound, CTA, or MRA. These imaging modalities allow for the visualization and measurement of blood flow patterns and velocities within the aneurysm^6–8^.

One important parameter that can be assessed is the aneurysm's filling status, which refers to the degree of blood flow into the aneurysm. Complete occlusion, or the absence of blood flow, is the desired outcome after endovascular coiling, as it reduces the risk of rupture and subsequent bleeding. Another important parameter is the stability of the coils within the aneurysm. Coils that are not tightly packed or are displaced from their intended position can result in incomplete occlusion and increase the risk of rupture^9–12^.

By evaluating blood hemodynamics inside ICA aneurysms, healthcare providers can make informed decisions regarding further treatment or follow-up, such as the need for additional coiling, surgical intervention, or regular monitoring to detect any changes in the aneurysm over time^13–16^.

It's worth noting that blood hemodynamics inside ICA aneurysms can be influenced by various factors, such as the size and shape of the aneurysm, the location within the brain, and the patient's overall health. Therefore, a thorough evaluation of the patient and the aneurysm is necessary to ensure that the best course of treatment is selected^17–20^.

Several factors can influence blood hemodynamics inside ICA aneurysms, including: aneurysm size and shape: the size and shape of the aneurysm can affect blood flow patterns and velocities. Larger aneurysms may have slower and more turbulent blood flow, which can make it more difficult to achieve complete occlusion with endovascular coiling. Aneurysm location: the location of the aneurysm within the brain can also affect blood flow. Aneurysms located at arterial bifurcations or other branching points may have more complex flow patterns, which can make it more difficult to achieve complete occlusion^21–24^. Aneurysm morphology: the morphology of the aneurysm, such as the presence of a neck or dome, can also affect blood flow. A wide-necked aneurysm may be more difficult to treat with endovascular coiling as it can be challenging to place the coils without compromising blood flow to surrounding vessels. Patient age and comorbidities: the patient's age and overall health can also affect blood flow. Patients with hypertension or other cardiovascular conditions may have altered blood flow patterns, which can impact the effectiveness of endovascular coiling. Treatment modality: the type of treatment used to manage the aneurysm can also affect blood hemodynamics. Endovascular coiling and surgical clipping both have their gains and difficulties, and the choice of treatment may depend on the specific characteristics of the aneurysm and the patient. By considering these factors, healthcare providers can make informed decisions regarding the most appropriate treatment plan for each patient. A thorough evaluation of the aneurysm and the patient's overall health is essential to ensuring the best possible outcome^25,26^.

If blood flow is still present inside the aneurysm after the endovascular coiling procedure, further intervention may be necessary to prevent rupture and potentially life-threatening hemorrhage. Therefore, evaluating blood hemodynamics inside ICA aneurysms is an important aspect of the management of these conditions.

A compelling situation comprising the interaction between the vessel wall and blood flow is atherosclerosis, which is a primary reason for death. Atherosclerosis happens primarily in positions of disturbed blood flow patterns. Another compelling pathology connected with most cardiovascular diseases is intravascular thrombosis in which near-wall transport becomes important^27^.

Due to the importance of the blood stream, there are several papers investigating the flow behaviors inside the aneurysm^28^. Recent works has extensively focus on the biomedical science via theoretical approach^29^. Meanwhile, several factors have been introduced to measure the hemodynamic parameters inside the aneurysm and predict the rupture of the aneurysm^16^. For these factors (i.e. wall shear stress, OSI, …), some critical limits are also suggested to avoid the bleeding of cerebral aneurysms. It is clear that the shape and size of the aneurysm are critical for the rupture of the cerebral aneurysm^30^. The main challenge is to connect these geometrical features to the critical range of the introduced parameters.

A vivo measurement of these factors is almost impossible for human cases and this motivates scholars to apply computational and theoretical approaches for the estimation of the hemodynamic factors^31,32^. Computational Fluid dynamic is a reliable and precise approach for the modeling of the blood stream and calculation of the hemodynamic factors. This technique is used for the simulation of different engineering problems.

This study tries to investigate the impacts of the sac section area and mean radius of the parent vessel on the risk of internal carotid artery (ICA) rupture. In addition, the influences of hematocrit and coiling porosity are investigated on 9 specific 1CAs. Transient NS equations are solved for the simulation of the blood stream inside the aneurysm^33^.

## Methods

The main data of aneurysm is acquired from Aneurisk website^34^. It is confirming that all methods were carried out in accordance with relevant guidelines and regulations. Besides, all experimental protocols were approved by of the Ca' Granda Niguarda Hospital and it is confirmed that informed consent was obtained from all subjects and/or their legal guardian(s). RSM statistical technique is used to select model in specific conditions. Then, CFD method is used for the simulations and analyze the flow hydrodynamic to find the high-risk region on the sac surface.

### RSM technique

In this study, we focus on investigating the impact of two geometric characteristics, namely the sac section area and the mean radius of the parent vessel, as well as two accessible features of blood, hematocrit (HCT), and coiling porosity, on the hemodynamics of the blood stream. To minimize the number of required simulations for the selected models and factors, we employ Design of Experiment (DOE) as our statistical method. By utilizing the Response Surface Methodology (RSM) with Central Composite Design (CCD), we are able to identify 25 specific run conditions for these factors and models, allowing for a more efficient analysis.

### Model selection

**Figure 1:**
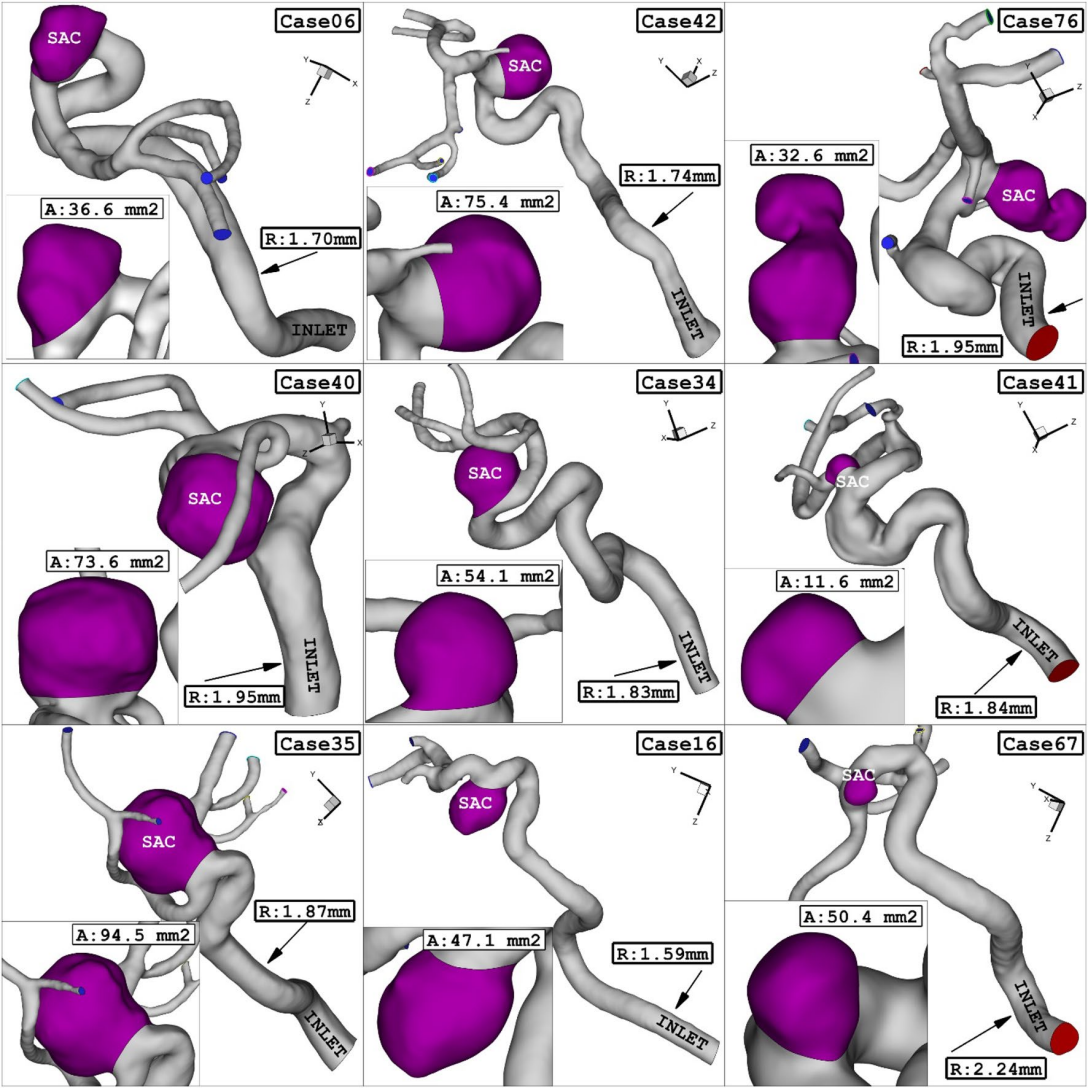
ICA aneurysm of 9 different cases.

In this study, we utilized nine real 3D models obtained from Aneurisk^34^. The geometries (.stl) of these models were used to conduct our analysis. In these chosen geometrical models, the sac section area ranges from 11.65 mm^2^ (case model 41) to 94.57 mm^2^ (case model 35), while the mean radius of the parent vessel varies between 1.59 mm (case 16) and 2.24 mm (case 67). The hematocrit (HCT) ranges from 0.35 to 0.5, and the coiling porosities range from 0.73 to 0.96^35,36^. Figure 1 provides a visual representation of the geometry of the selected models, showcasing their geometrical characteristics.

### Computational modeling

In this study, we simulate the blood flow inside the vessel by solving the transient Navier–Stokes equations. We assume that the blood flow is incompressible, non-Newtonian, and laminar. Computational fluid dynamics (CFD) approach is commonly used for simulating engineering problems^37–41^. In this work, we employ a CFD technique with a simple algorithm to simulate the blood flow within the vessel. The viscosity of the blood is determined using the Casson model^37^.

**Figure 2:**
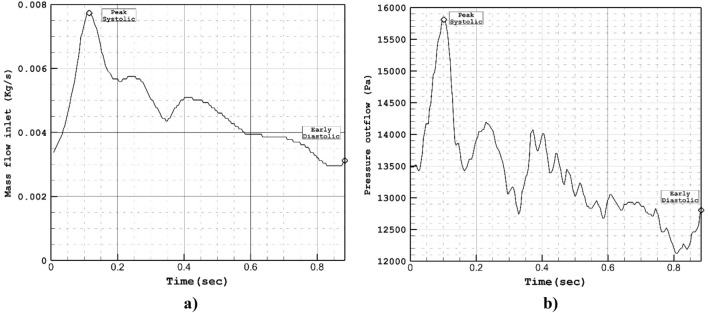
Applied (**a**) mass and (**b**) pressure profile at inlet and outlets.

To model the pulsatile blood flow entering the parent vessel, we apply a pressure profile, which is plotted in Fig. 2. This figure also displays the mass flow rate at the inlet corresponding to the applied pressure profile. To ensure a fully converged solution, we solve three cycles of the blood flow, as depicted in Fig. 2^42,43^. The applied pressure profile exhibits four distinct stages, representing critical time points. We set the initial condition based on t = 0 s on the cardiac cycle.

**Figure 3:**
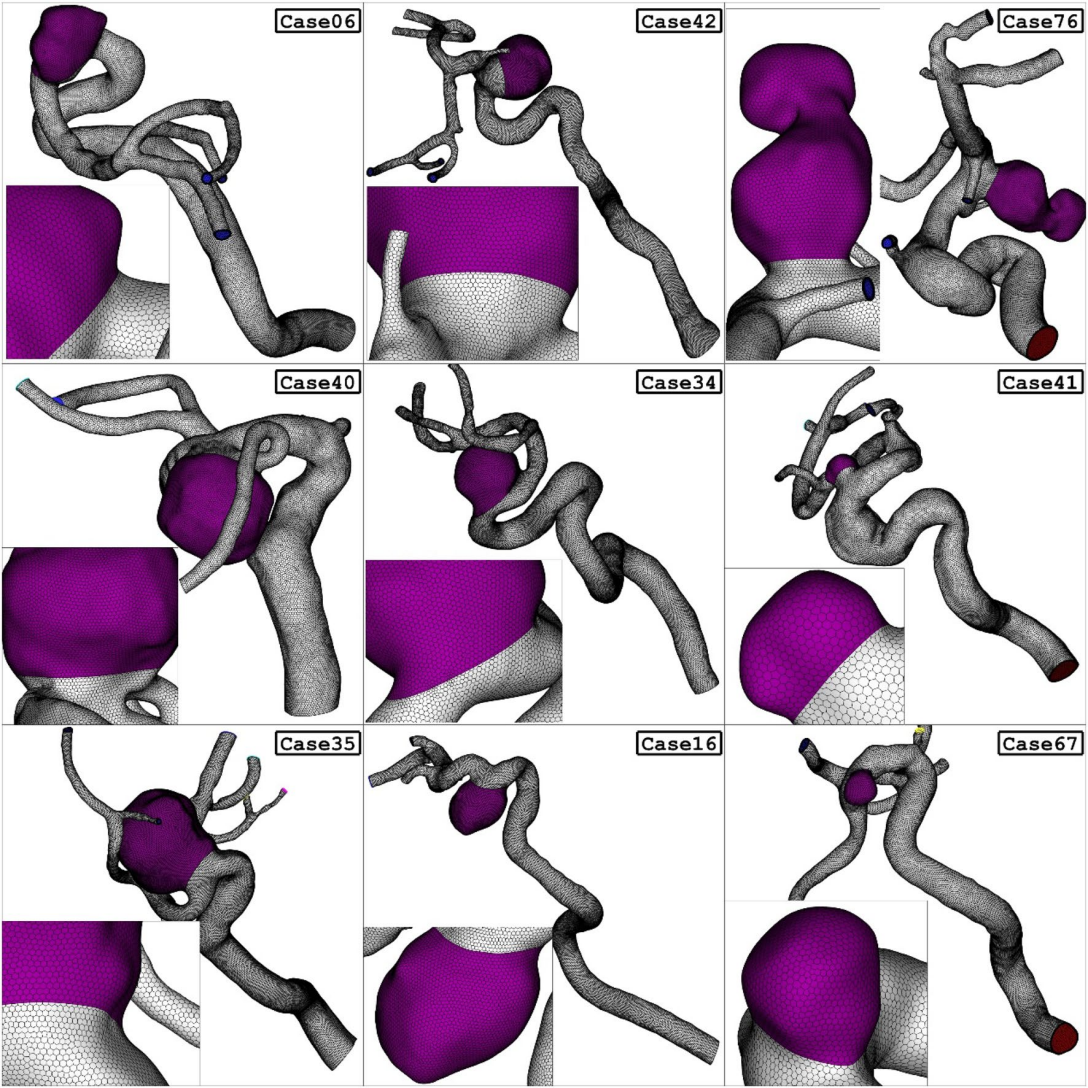
Grid generation.

Figure 3 demonstrates the close-up view of the size and shape of the used grid for one of the selected models. To decrease the grid size near the wall of the aneurysm, the boundary layer is used to improve the resolution of the grid. The convergence of the results is evaluated by the residual of the main governing equations and it was less than 10e-4 for this study^44,45^. The computational technique is extensively used for advance the mechanical system^46–50^.

## Results and discussion

### Pressure

**Figure 4:**
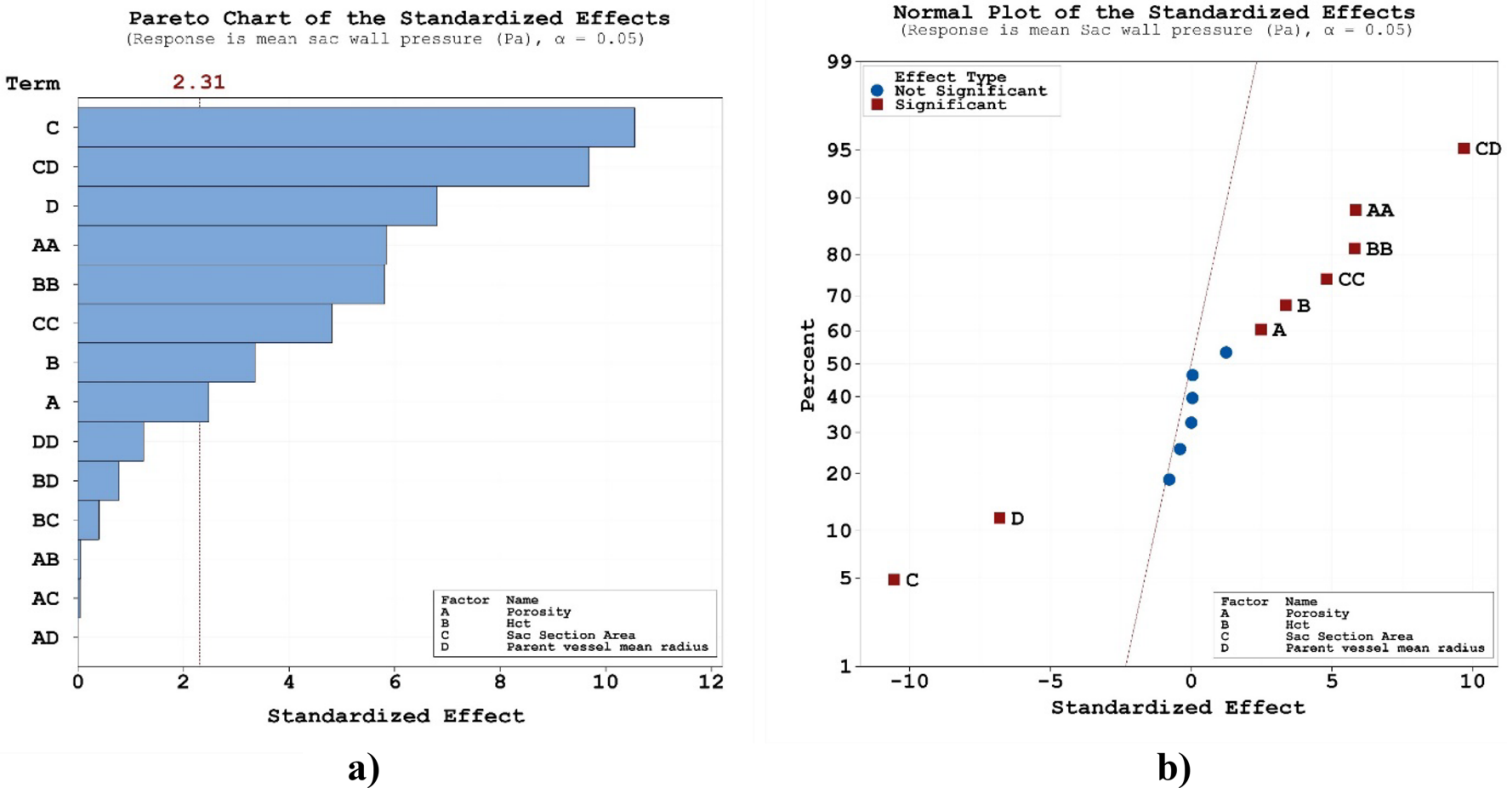
(**a**) Pareto chart, (**b**) normal plot of mean sac wall pressure at peak systolic.

Present study also tries to reveal the impacts of the geometrical feature of the aneurysm on pressure distribution on sac surface. RSM is applied on mean pressure on sac wall and results are presented in 4a. The obtained results confirm that all of selected parameters (porosity, HCT, Sac section area and mean radius of parent vessel) is effective on the mean pressure value on the sac wall. Normal plot (Fig. 4b) also indicates that blood HCT and coiling porosity have positive effects on the pressure value while sac section is and mean radius of parent vessel have negative impacts on mean pressure.

**Figure 5:**
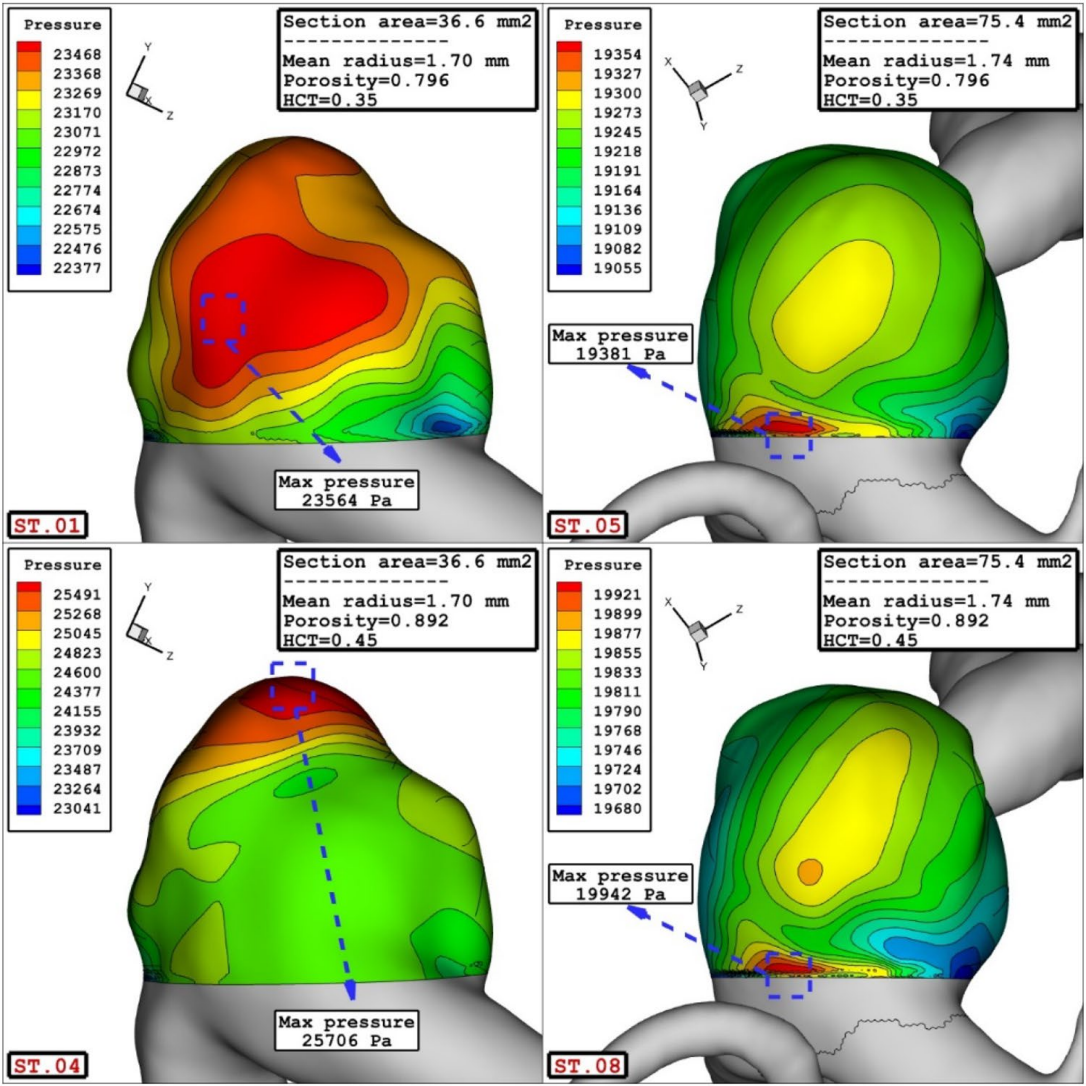
Effect of sac section area on mean sac wall pressure.

Figure 5 illustrates the influence of sac section area on the mean pressure on the sac wall. Comparison of these cases show that the increasing sac section area from 36.6 to 75.4 mm^2^ results in 20% pressure reduction on the sac wall.

### Average velocity

**Figure 6:**
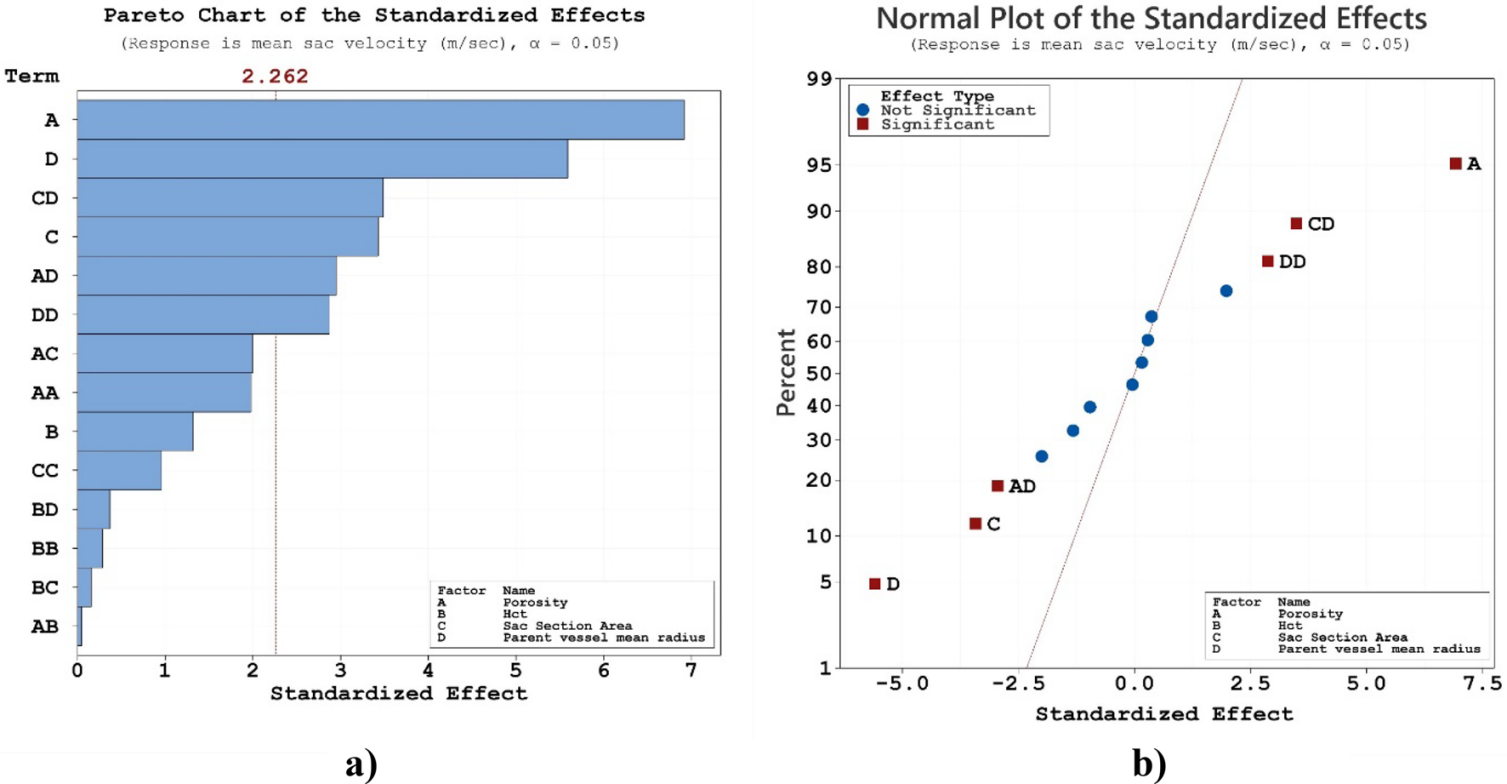
(**a**) Pareto chart, (**b**) normal plot of mean sac velocity at peak systolic.

**Figure 7:**
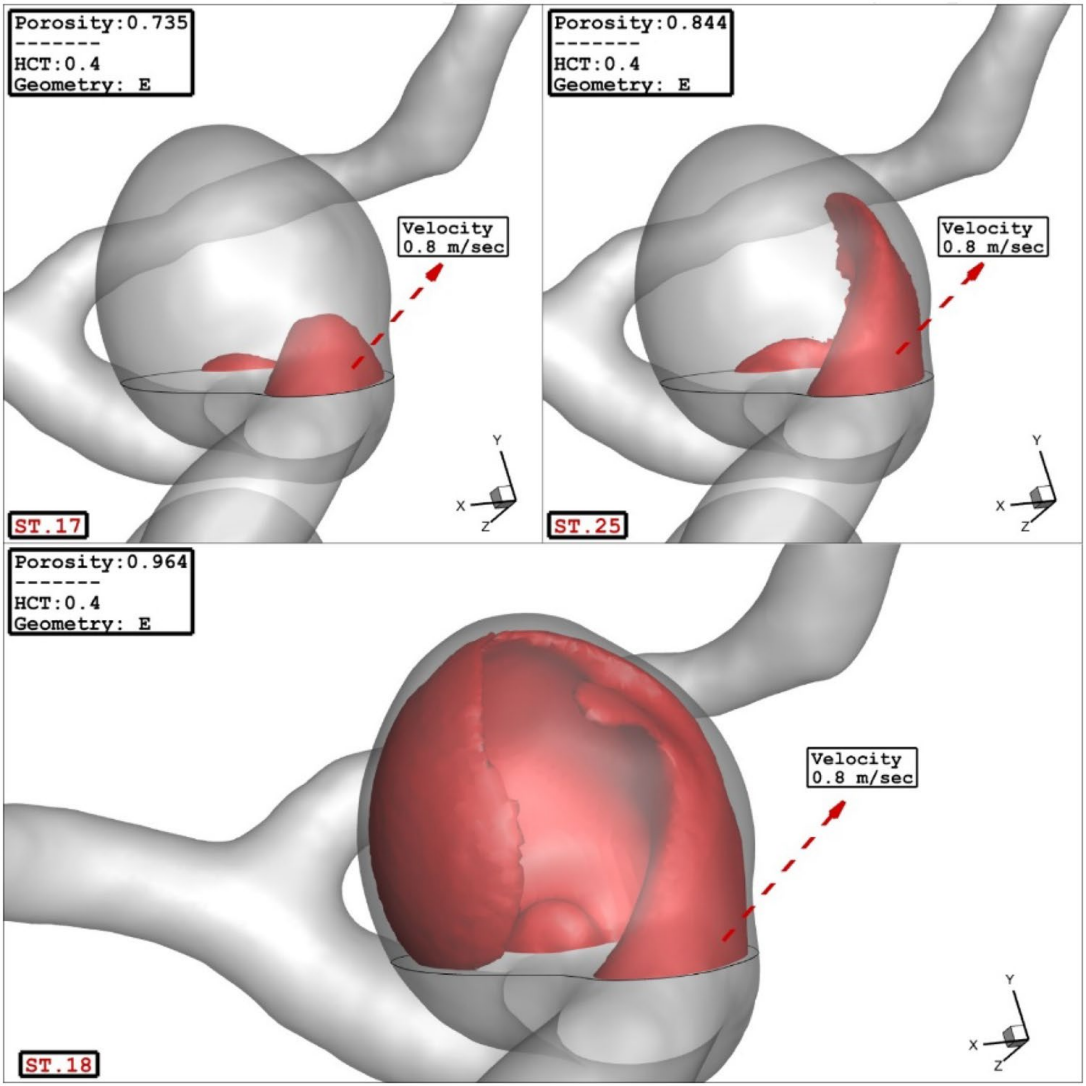
Effect of porosity on mean sac velocity at peak systolic.

In present work, the effects of the selected parameters (porosity, HCT, Sac section area and mean radius of parent vessel) on the average velocity inside the sac are also investigated. As shown in Fig. 6a, all parameters (excluding HCT) have meaningful impacts on the average velocity on the sac wall. Normal plot (Fig. 6b) of mean average velocity indicates that the coiling porosity and mean radius of the parent vessel have great positive and negative impacts, respectively, on the average velocity value inside the aneurysm. The role of coiling porosity on the velocity variations inside the aneurysm is displayed in Fig. 7. In specific condition, the decreasing the coiling porosity restricted high blood velocity inside the aneurysm. It is also observed that the high velocity region occurs near the aneurysm wall.

**Figure 8:**
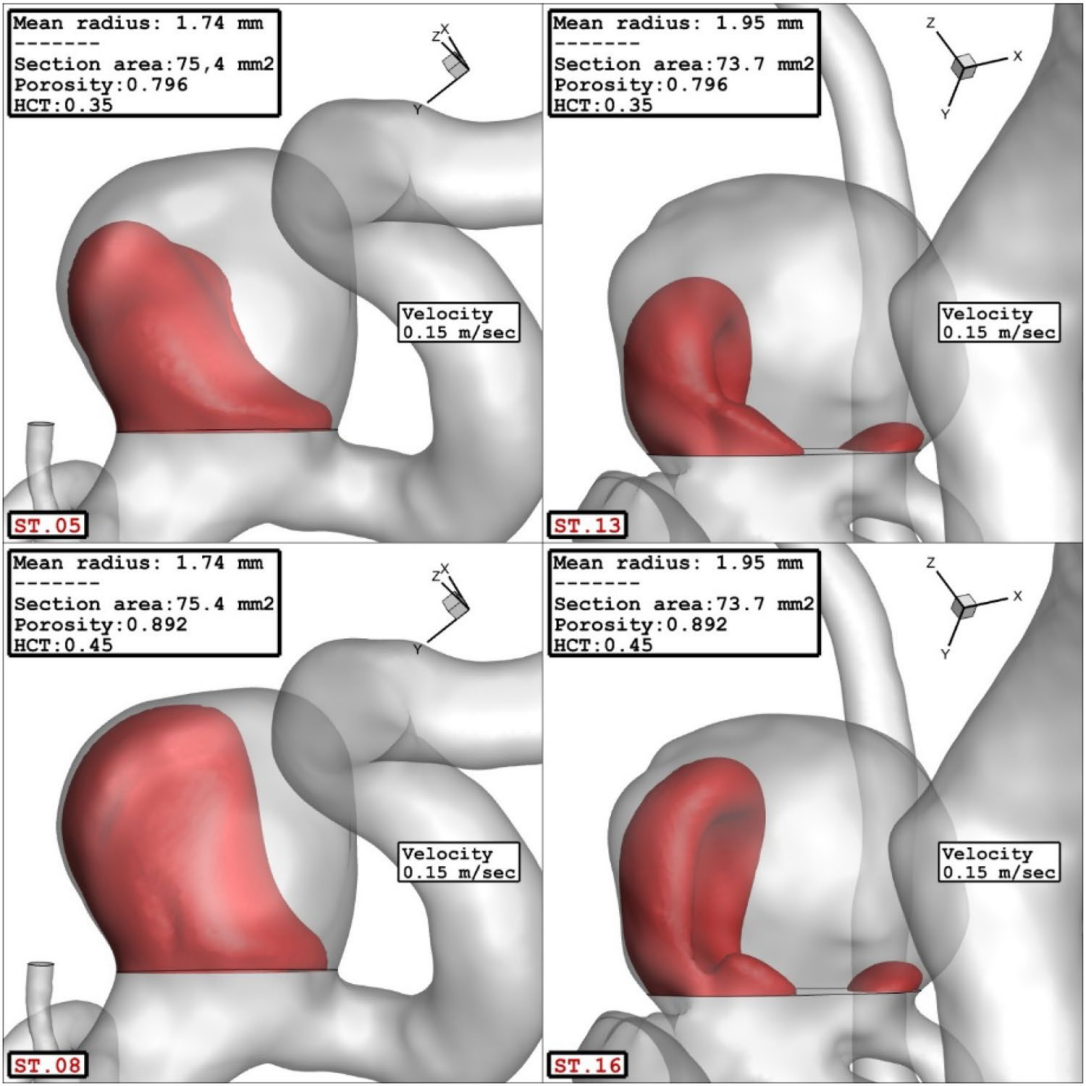
Effect of parent vessel mean radius on mean sac velocity at peak systolic.

**Figure 9:**
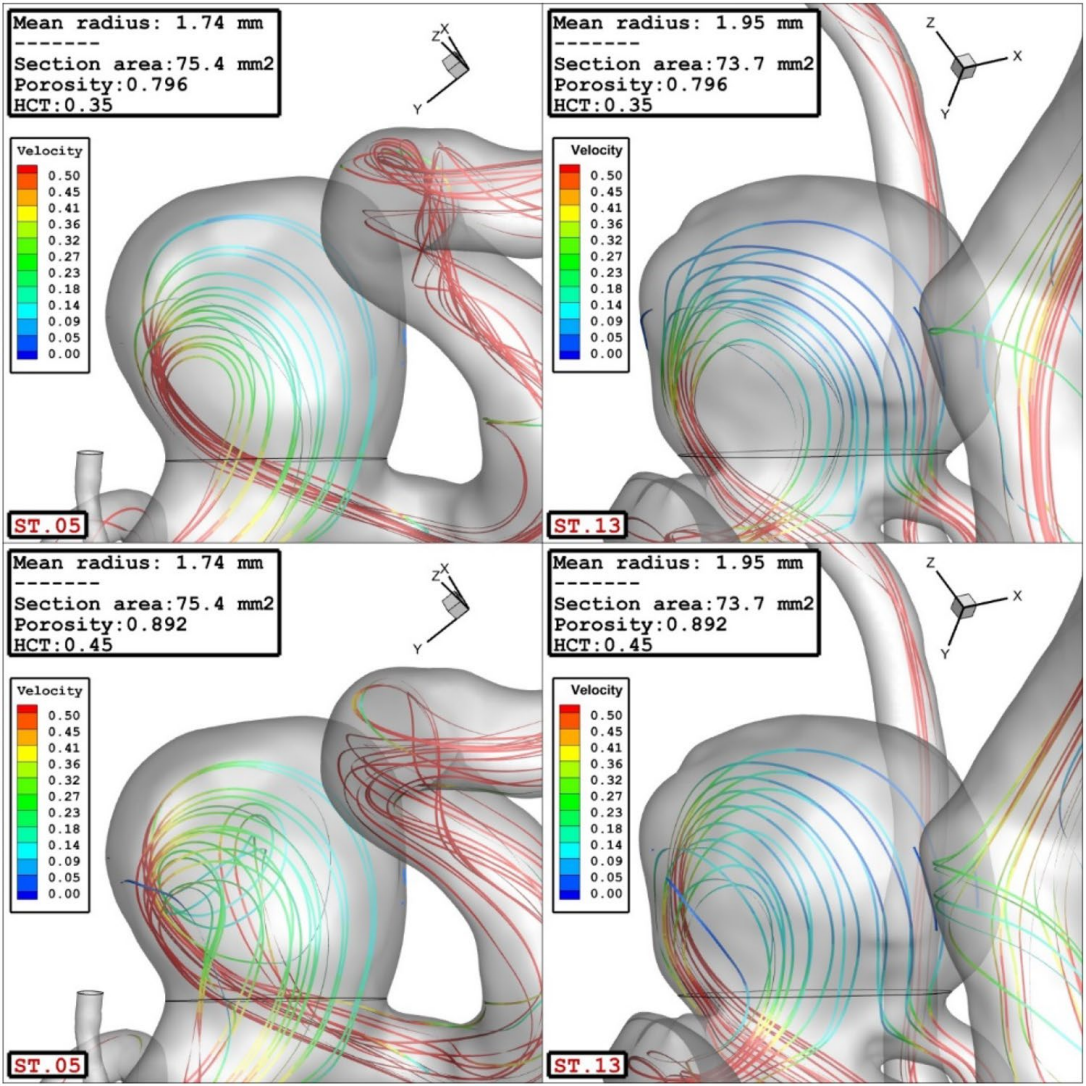
Effect of parent vessel mean radius on streamlines at peak systolic.

The impacts of the mean radius of parent vessel on the mean average velocity inside the sac are illustrated in Fig. 8. Achieved results show that increasing the radius of parent vessel declines the velocity inside the sac domain. Meanwhile, increasing the blood hematocrit also increase the velocity of the blood stream inside the sac. Figure 9 demonstrates the velocity variations on the streamline for cases with different parent mean radius of 1.74 mm and 1.95 mm.

**Figure 10:**
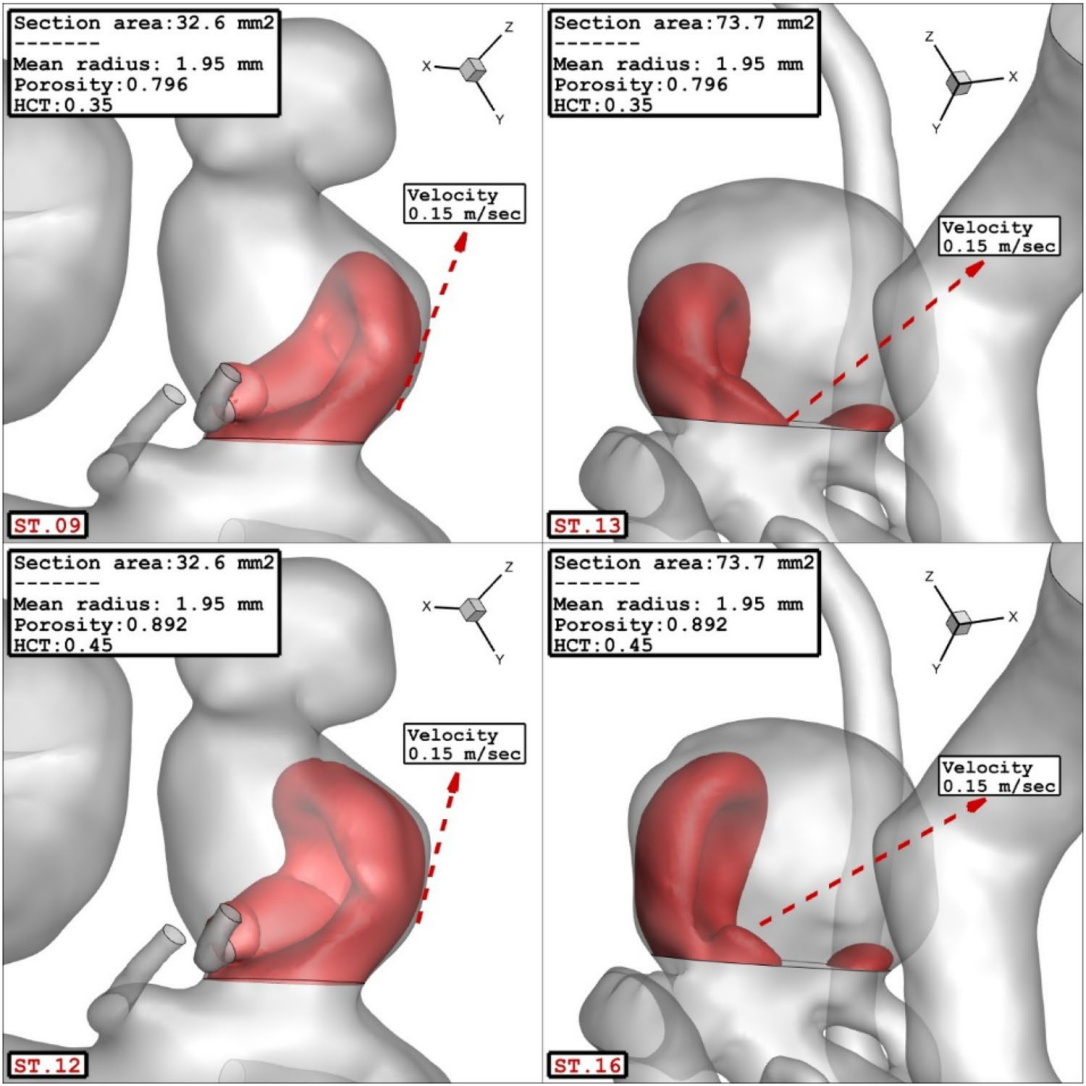
Effect of sac section area on mean sac velocity at peak systolic.

**Figure 11:**
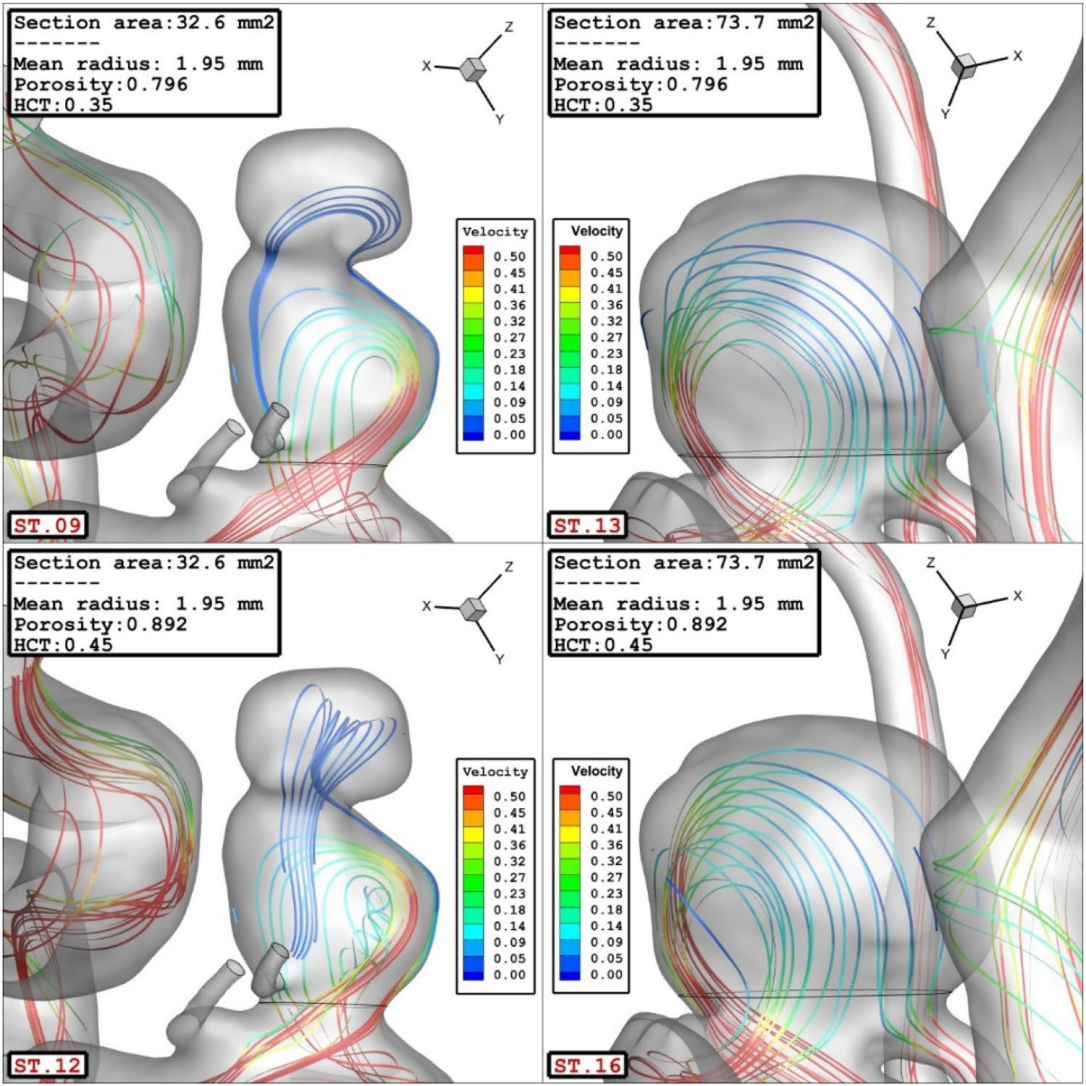
Effect of sac section area on streamlines at peak systolic.

The influence of the sac surface area on the blood velocity and structure are presented in Fig. 10. The comparison of the velocity iso-surface (v = 0.15 m/s) show that the velocity of the blood inside the aneurysm is higher in the cases with lower sac section area. The contour of the streamline colored by the velocity (Fig. 11) confirm that blood velocity decreases after the first interaction with sac wall and circulation is more intense near the neck of aneurysm.

## Conclusion

This article presents a comprehensive computational study aimed at investigating the impact of various parameters on the hemodynamics of blood flow within an ICA aneurysm. The parameters under investigation include coiling porosity, HCT, sac section area, and mean radius of the parent vessel. Computational fluid dynamics is employed to analyze the blood flow dynamics by solving transient N–S equations. A total of 9 specific ICA geometries are examined across 25 different scenarios, encompassing various geometric and operational conditions. To streamline the analysis, a response surface model is utilized to identify specific run conditions for the selected parameters, reducing the number of required simulations. The study thoroughly explores the effects of these parameters on key hemodynamic characteristics associated with aneurysm rupture, including pressure and average velocity. Additionally, the blood flow patterns are visually depicted and compared under different conditions to ascertain the underlying physical mechanisms related to these parameters. Achieved results show that the increasing sac section area from 36.6 to 75.4 mm^2^ results in 20% pressure reduction on the sac wall. Achieved findings confirm that the velocity of the blood inside the aneurysm is higher in the cases with lower sac section area.

## Data Availability

All data generated or analysed during this study are included in this published article.
